# The Pathway to Achieving Universal Health Coverage in the Democratic Republic of Congo: Obstacles and Prospects

**DOI:** 10.7759/cureus.41935

**Published:** 2023-07-15

**Authors:** Moussa Issa

**Affiliations:** 1 Department of Emergency Medicine, Calderdale & Huddersfield National Health Service (NHS) Foundation Trust, Huddersfield, GBR; 2 Department of Health Research, Lancaster University, Lancaster, GBR

**Keywords:** democratic republic of the congo, health insurance in the drc, health expenditure, healthcare system, universal health cover

## Abstract

This paper explores the complexities surrounding achieving universal health coverage (UHC) in the Democratic Republic of Congo (DRC) and proposes viable strategies to overcome the obstacles. The study's findings contribute to the global discourse on UHC in resource-limited settings and hold significant implications for policy formulation and implementation in both DRC and similar contexts. The introduction emphasises the importance of UHC in promoting equitable access to quality healthcare services for all individuals.

Nevertheless, the DRC faces numerous challenges on its path to UHC. This paper identifies four key challenges: Firstly, the fragile healthcare infrastructure in the DRC necessitates the establishment of better-equipped facilities, an adequate healthcare workforce, and improved access to essential medical supplies. These factors hinder the provision of comprehensive health services and impede progress towards UHC. Secondly, socio-economic barriers such as persistent poverty, income disparities, and regional variations pose significant obstacles to achieving UHC in the DRC. Limited financial resources and widespread poverty prevent individuals from accessing healthcare services, exacerbating health inequities. Thirdly, weak health governance, inadequate policy implementation, and limited coordination among stakeholders impede the effective delivery of healthcare services in the DRC. Thus, strengthening governance structures and enhancing policy implementation are essential for UHC. Lastly, the absence of comprehensive health information systems and poor data management hinder evidence-based decision-making and resource allocation. Addressing these deficiencies is vital for monitoring progress and guiding policy formulation towards UHC.

Given these challenges, this paper proposes potential solutions and future perspectives for achieving UHC in the DRC. These include strengthening health systems, implementing social protection mechanisms, enacting policy reforms, enhancing governance structures, and strengthening health information systems. Investments in robust health information systems, data collection and management improvements and the enhancement of capacity for health research and surveillance facilitate evidence-based decision-making and progress towards UHC.

In conclusion, the DRC faces obstacles related to healthcare infrastructure, socio-economic factors, governance issues, and deficiencies in health information systems in its pursuit of UHC. However, by addressing these challenges through targeted interventions, policy reforms, and improved governance, the DRC can make strides towards ensuring equitable access to high-quality healthcare for all its citizens. Collaboration between national and international stakeholders is crucial for sustaining progress towards UHC and promoting health equity within the country.

## Editorial

Introduction 

Providing adequate and high-quality healthcare, free from discrimination based on race, politics, or religion, is a fundamental human right. This right has been recognised and protected internationally since 1948 through the Universal Declaration of Human Rights [1] and the International Covenant on Economic, Social, and Cultural Rights of 1966 [2]. The World Health Organization (WHO) defines universal health coverage (UHC) as a principle that ensures everyone has access to necessary and effective health services of acceptable quality without suffering financial hardship [3-5].

The concept of UHC was initially introduced by the WHO in 1970, supporting the "health-for-all agenda." This agenda laid the groundwork for the seminal Primary Health Care (PMC) Conference held in Alma Ata in 1978 [6], which set goals and resolutions, including UHC [7,8], to achieve 'health for all'. In 2012, the United Nations (UN) urged countries to incorporate UHC into future discussions [9]. Subsequently, in 2015, UN members agreed upon the Sustainable Development Goals (SDGs) and established ambitious plans for a safer, more equitable, and healthier world by 2030. Target 3.8 of the SDGs focuses explicitly on UHC [10,11].

Evidence indicates that expanded health coverage improves access to care and health outcomes, particularly for the most disadvantaged populations [12]. The primary objective of UHC is to alleviate the financial burden associated with healthcare provision, preventing individuals from incurring high out-of-pocket costs that can exacerbate poverty among already impoverished households [12]. Access to health services enables individuals to actively contribute to society by facilitating education for young people and enabling adults to work and earn income. Financial risk protection prevents individuals from experiencing hardship when they have to pay for healthcare costs out of their pockets in the event of severe illness. Therefore, UHC not only addresses the improvement of health (SDG 3.8) but also promotes other SDGs such as poverty reduction (SDG1), nutrition (SDG2), education (SDG4), gender equality (SDG5), economic growth, and job creation (SDG8) [13].

In April 2001, during the Abuja Declaration, heads of state of African Union countries committed to allocating at least 15% of their budget to improving the health sector [14]. However, UHC's achievement heavily relies on a nation's economic and socio-political stability. Despite the commitment of African countries to UHC, affordable access to healthcare remains elusive for the population of sub-Saharan Africa. In many countries, basic healthcare is unaffordable for numerous families. Non-communicable diseases and injuries account for over 30% of the disease burden in low- and middle-income countries (LMICs), including the Democratic Republic of Congo (DRC) [15]. These conditions cause nearly 800,000 deaths annually among individuals under 40, surpassing the combined mortality rates of HIV, tuberculosis, and maternal diseases [16].

In January 2019, the DRC witnessed a peaceful power transfer between outgoing and newly elected presidents [17], rekindling hope after decades of misery. The current administration is committed to providing basic healthcare to the entire population, as reflected in the Head of State appointing a dedicated cabinet responsible for UHC. This cabinet's role is to assess the country's progress towards UHC to benefit all Congolese citizens.

This article aims to analyse the challenges faced by the DRC on its path to achieving UHC and evaluate the country's capacity to implement UHC for its entire population.

The DRC's context

Geographically, the DRC occupies the largest land area in sub-Saharan Africa, surpassing Eastern Europe in size [17]. Additionally, it shares borders with nine neighbouring countries [18]. The DRC had an estimated population of approximately 89,561,404 in 2020, characterised by a predominantly youthful demographic [19]. The overall figure at birth was about 60.6 years in 2019, with men having an estimated life expectancy of 59.1 years and women 62.2 years [20]. Conversely, the infant mortality rate was 66.1 per 1,000 live births in 2019 [21].

From an economic standpoint, the DRC houses the third-largest impoverished population globally. Nevertheless, it possesses unique natural resources, including rare minerals, significant hydropower potential, expansive arable land, extensive biodiversity, and the second-largest rainforest [17]. The country holds the potential to emerge as one of Africa's most prosperous economies if issues of poor governance and corruption are effectively addressed. However, it is disconcerting that poverty levels persist at a high rate. In 2018, the World Bank estimated that approximately 73% of Congolese individuals lived on less than $1.90 daily, signifying extreme poverty [17,22]. This translates to approximately one in six people in the DRC experiencing severe poverty within sub-Saharan Africa [17].

Moreover, the DRC grapples with persistent conflicts in its eastern territory, often fueled by the activities of armed groups, leading to ongoing insecurity. In addition to these security crises, the country faces recurrent epidemics such as cholera, measles, and Ebola. The 11th Ebola Virus Disease (EVD) epidemic was officially declared concluded on November 18, 2020, claiming the lives of 55 individuals [23]. These circumstances have detrimentally impacted the country's healthcare system and overall health indicators.

Regarding social indicators, the DRC ranks 175th out of 189 countries on the 2020 Human Development Index [17], with a human capital index of 0.37%. This rate falls significantly below the average of 4.0 observed among other countries in sub-Saharan Africa [17]. Consequently, a child born in the DRC today would only be 37% as productive as they could potentially be as an adult if they had received an excellent education and experienced good health during their early years. Additionally, the prevalence of malnutrition among Congolese children is estimated to be 43% [24].

In epidemiological terms, the recent coronavirus disease 2019 (COVID-19) crisis has highlighted the impact of global population movement, trade, and food exchange on transmitting infectious diseases [13]. In the DRC, several communicable diseases are resurgent with epidemic potential, further exacerbating the incidence of non-communicable diseases. This dual burden of communicable and non-communicable diseases significantly contributes to mortality and morbidity rates within the country. The WHO reports that non-communicable diseases account for 40 million deaths annually worldwide (representing 71% of all global deaths, with 77% occurring in LMICs). In 2016, the WHO estimated that non-communicable diseases constituted 28% of mortality in the DRC [25].

Despite well-intentioned efforts, the primary challenge faced by LMICs remains the need for more financial capacity to ensure universal access to primary healthcare [26]. Sluggish advancements, economic challenges, mismanagement, and corruption have hindered progress towards achieving UHC in the DRC. Inadequate health coverage restricts the country's ability to implement a primary healthcare strategy, a pivotal aspect of health since the Astana Declaration, which aims to accelerate progress towards UHC [15]. In 2014, the country endorsed policy and legislative recommendations to decentralise the health system and reform the healthcare sector and then presented a new draft law on UHC in 2016 [22].

Universal health care and the DRC today

Funding for UHC in the DRC

UHC advancement relies on the WHO health financing framework, emphasizing sufficient service coverage and financial protection [27,28]. Adequate health financing requires the involvement of the government budget, private payments, and direct service delivery by non-governmental organisations [26]. To prevent unnecessary mortality, alleviate suffering, and minimise the risk of catastrophic health expenditures, UHC should be funded through various means, such as revenue from the state budget, mandatory or voluntary prepaid insurance, direct out-of-pocket payments by users, and external assistance. Additionally, pooling funds, purchasing services, and co-payments for essential services are important components of this financing strategy [16,28].

Extensive evidence demonstrates that heavy reliance on direct payments exposes health service users to severe financial hardships [22,29]. A household is considered to experience catastrophic expenditure if it spends more than 10% of its annual health expenditure. In the DRC, similar to other LMICs, healthcare expenses are primarily borne by households, posing a significant barrier to achieving UHC. The country faces economic constraints and is plagued by corruption and mismanagement. Direct payments, averaging between US$ 4.8 and 10, have predominantly contributed to the impoverishment of Congolese households [30]. Co-payments constituted the primary source of health financing in 2014, accounting for 42% of the total health expenditure. The poorest households, comprising nearly 64% of the DRC population, bear these costs. However, according to the most recent data from 2012, 4.8% of households experience catastrophic health expenditures [15].

Moreover, 34% of individuals who fell ill within the past six months did not seek healthcare, with 35% attributing financial difficulties as the primary reason for not accessing health facilities [15]. This alarming data reveal that nearly 20% of Congolese households face financial barriers when obtaining health services. The national budget allocation to the health sector reflects the low priority given to healthcare in the DRC. In 2014, only 6.9% of the country's budget was allocated to the health sector, and this share dropped to approximately 6% in 2017 [15].

Furthermore, the DRC heavily relies on international aid. Between 2008 and 2014, foreign aid accounted for nearly 40% of the total funding for the health sector. Private funding is minimal, contributing only 4% to the overall health expenditure in 2014. Non-governmental organizations and national charitable foundations accounted for 1% of the total health expenditure in the same year. The pooling of resources is virtually nonexistent, with numerous small and mostly voluntary funds providing inadequate financial security. Funds designated for the needy and vulnerable are fragmented [15].

Social Determinants of Health and Health Inequalities

The initial step towards achieving adequate UHC entails addressing the social determinants of health. While the health sector is responsible for UHC, favourable outcomes necessitate collaboration across multiple sectors [13]. These outcomes are influenced by various internal and external factors within the health system. Poverty, for instance, exerts a detrimental impact on health outcomes by impeding access to healthcare, compromising the quality of food supply, limiting the choice of residential locality, compromising water and sanitation quality, increasing exposure to harmful substances such as alcohol and drugs, restricting access to health literacy and education, undermining social status, and fostering psychosocial and physiological stress [26].

Several countries have successfully implemented UHC in recent decades, reducing population inequalities. However, even in higher-income nations, marginalised communities face exclusion, enduring higher poverty rates and experiencing poorer health statuses and outcomes than their more affluent counterparts. LMICs face a particularly dire situation. In the DRC, efforts to address health determinants have yielded unsatisfactory results. According to the Ministry of Health DHS-RDC II report (2013-2014), households accessing drinking water from an improved source increased from 46% in 2007 to 49% in 2013. However, this increase was unevenly distributed, rising from 24% to 32% in rural areas and from 80% to 85% in urban areas. Only 18% of households have access to improved sanitation facilities (21% in urban areas and 17% in rural areas). Merely 14% of households have access to electricity (42% in urban areas compared to 0.4% in rural areas). The illiteracy rates among individuals aged six and above remain alarmingly high, with 19% of women and 8% of men lacking formal education. Nationally, 43% of children aged 0-59 months suffer from chronic malnutrition, while 23% experience severe malnutrition. Additionally, 8% suffer from acute malnutrition and 3% from the severe form; 23% are underweight, and 7% experience severe underweightness [24].

The healthcare system and pitfalls to UHC in the DRC

Drawing on the scholarly works titled "Healthy Systems for Universal Health Coverage - A Shared Vision for Healthy Lives" [13] jointly published by the WHO and the World Bank, and the report titled "National Strategic Plan for Universal Health Coverage 2020-2030" [15] issued by the Government of the DRC, the present analysis endeavours to evaluate the country's level of preparedness in implementing UHC for its populace. Establishing a dependable UHC system necessitates a resilient health system, encompassing both public and private entities, institutions, and resources to enhance, preserve, and restructure health-related services [13,31]. Moreover, the WHO has provided recommendations for assisting organisations in constructing health systems that effectively tackle contemporary challenges based on the fundamental building blocks for health systems [32]. Consequently, constructing a robust health system is paramount in attaining UHC objectives [13]. In Tanzania, health insurance initiatives introduced during the mid-1990s extend coverage to approximately 30% of the population. In an exploratory study conducted in a rural region, wherein the experiences and perspectives of individuals aged 60 years and above were examined, it was discovered that while the respondents expressed gratitude for the accessibility of healthcare services, their primary concerns revolved around the scarcity of essential medications and rudimentary check-ups, prolonged waiting times, limited referral services, deficiencies in infrastructure quality, confidentiality concerns, and issues about personal autonomy [33]. Although this study was conducted in a similar setting to the DRC, it is important to note that participants were not randomly selected, which may limit its applicability outside their natural setting. Nevertheless, the study illuminates the intricate dynamics inherent within a health system, encompassing the interplay between service providers and beneficiaries [33].

Service Delivery

The Government of the DRC recently published a report on UHC from 2020 to 2030, comprehensively analysing the nation's challenges and social determinants. Regrettably, the report lacks a clear strategy that articulates the means to achieve effective service delivery. It is widely acknowledged that service delivery plays a pivotal role in bridging the divide between the health system and the population. Consequently, to effectively promote UHC, health systems must prioritise five fundamental performance characteristics, namely resilience, quality, efficiency, equity, and responsiveness, as stipulated by the Organisation for Economic Co-operation and Development (OECD) in 2002 and 2015 [34,35]. Efficiency and equity in health service delivery require a greater focus on services at the grassroots level, mainly primary health care (PHC) [36]. Nevertheless, given the prevailing economic circumstances, inadequate health infrastructure, and the inability to initiate PHC in the DRC, the implementation of UHC in the country appears to be beyond reach.

Health Workforce

The health workforce encompasses individuals in private and public sectors, working full-time or part-time and stationed at a single workplace or multiple locations. These individuals voluntarily contribute to the healthcare sector [32]. While the WHO has recommended a ratio of doctors to the population of 1:1,000 [37], the DRC government's report cites 30,700 registered doctors (for 85 million people) and 115,900 nurses. This figure of 0.03 doctors (per 1000 population) not only contradicts the data published by the World Bank (per 1000 population) of 0.4 in 2018 but needs to be revised to meet the workforce needs to be required for adequate UHC [38]. The ability of a government to achieve its health objectives depends primarily on the skills, knowledge, motivation, and commitment of capable personnel involved in managing and delivering health services [32]. However, insufficient incentives for healthcare workers can promote unsafe practices, unfavourable attitudes towards patients, and non-compliance with recommendations, which can further exacerbate the issue of brain drain and impede the return of those trained abroad [39]. In the face of this reality, the DRC is far from moving closer to successful national health care.

Health Information Systems

The health information system oversees health data management, encompassing the generation, compilation, analysis, synthesis, and assurance of data's overall quality, relevance, and timeliness. It transforms these data into information crucial for decision-making [32]. National governments should set and adjust their priorities based on the best available local data [16]. Unfortunately, the information system in the DRC remains reliant on paper-based records and lacks integration with individual computers. Consequently, obtaining accurate data poses a challenge [15]. The country's poor internet infrastructure further hinders the immediate transition to a digitised health system that would facilitate real-time information collection, analysis, dissemination, and decision-making. This represents another obstacle to the implementation of UHC in the DRC.

Access to Essential Medicines

Access to essential medicines necessitates ensuring product quality through robust monitoring and regulatory systems supported by appropriate legislation and governance structures within the public sector. Additionally, a robust supply chain is required to prevent the circulation of substandard and counterfeit medicines. Furthermore, adequate procurement or reimbursement systems should be established to minimise out-of-pocket payments. A few years ago, a scoping review of five middle-income countries (Ecuador, Ghana, the Philippines, South Africa, and Ukraine) assessed how UHC-related regulations promote the affordability and financing of essential medicines [40]. The review highlighted that in Ecuador, consumers have a legal right to access the retail prices of medicine labels. At the same time, in the Philippines, pharmaceutical companies must indicate the retail prices, which cannot exceed the maximum retail price. South Africa has implemented stricter regulations, providing consumers with details of the single exit price, availability, pricing mechanism, supply chain, distributors, retailers, and wholesale prices. In Ukraine, individuals have the right to be informed about the medicines they are entitled to under the medical guarantee program [40]. The economic context of these countries, although not directly comparable to that of the DRC, exemplifies the measures undertaken by certain nations to implement UHC for their respective populations. In order to effectively implement UHC in the DRC, substantial endeavours must be made to organise and regulate the pharmaceutical sector, supported by solid legislation aimed at reducing the prevalence of counterfeit medications. The repercussions of drug counterfeiting in the DRC are of great concern, as the supply chain for essential medicines remains unclear, inadequately regulated, and susceptible to various forms of misuse.

Financing

Domestic resource mobilisation is crucial for advancing towards UHC [13]. The Addis Ababa Action Agenda emphasises that every country must take responsibility for its socio-economic development by promoting domestic sources of finance as the primary mechanism to meet resource requirements for achieving the Sustainable Development Goals [41]. Successful implementation of UHC necessitates strengthening the financing process through legislative measures. For instance, the proposed National Health Insurance (NHI) scheme in South Africa proposes funding through prepayment taxes from the general treasury, supplemented by mandatory payroll and surcharge taxes [42]. However, previous attempts to implement NHI in South Africa faced various challenges, including higher unemployment rates, diminished trust in state institutions, conflicting political intentions, corruption, independent health insurance schemes, and multiple epidemics [43]. In the DRC, policymakers must establish alternative financing mechanisms and reduce dependence on international aid by implementing a progressive taxation system that generates sufficient domestic funding. Additionally, expanding community-level pooling arrangements, where participating families pay a predetermined membership fee in exchange for a certain level of care packages, can contribute to achieving UHC [44]. Managers and leaders must be accountable and ensure the responsible allocation of funds for their intended purposes.

Leadership/Governance

Governance is critical to the emergence of any health system [13], and its plan must include international coordination and partnership [34]. Governance should be grounded in evidence-based national health strategies involving all stakeholders, such as the Ministry of Health, civil society, service providers, private pharmacies, large companies, and citizens. Rwanda serves as an exemplar of significant progress in improving its health system. In response to a post-genocide context characterised by a shortage of health workers, inequality, and poor quality of care in health facilities, Rwanda launched "Vision 2020" to stimulate economic growth and recovery and reduce external aid [45]. The country improved its infrastructure, addressed corruption issues [39] and gradually increased the budget for health [46]. Rwanda actively took on the responsibility of donor coordination, implementing an aid effectiveness monitoring mechanism and a robust financial management system. Additionally, the country implemented substantial salary increases for healthcare workers and introduced performance-based incentives to enhance staff retention. The remarkable dedication displayed by Rwanda's leadership has yielded favourable outcomes for the health of its population. To achieve comparable advancements, the government of the DRC must prioritise good governance, refrain from utilising national projects for political gain, ensure the appointment of competent individuals to appropriate positions, avoid the distribution of public functions based on political alliances, and cultivate an environment conducive to such progress.

Conclusion

The implementation of UHC in the DRC faces significant challenges in the current context. However, the gradual implementation of UHC is feasible, taking into account various factors that include geographical, political, socio-cultural, economic, and organisational dynamics. To effectively implement UHC in the country, the government must demonstrate an unwavering commitment to improving the regulatory framework, ensuring sufficient domestic funding, investing in a greater number of competent health professionals, improving patient safety and quality of health care, including and protecting vulnerable and marginalised populations, and adopting an equitable, non-discriminatory and human rights-based approach. In addition, immediate attention must be given to strengthening the health system and expanding access to essential medicines and health technologies. Before the implementation phase begins, it is crucial to improve cooperation between the government and national and international partners, strengthen transparency and accountability mechanisms, strengthen the judicial system to fight corruption, and address the social determinants of health. Neglecting these pivotal aspects would reduce this vision to merely demonstrating good intentions and a vacuous political catchphrase.
